# Depression and anxiety disorders in patients with multiple sclerosis: association with neurodegeneration and neurofilaments

**DOI:** 10.1590/1414-431X202010428

**Published:** 2021-01-15

**Authors:** C.B. Tauil, A.D. Rocha-Lima, B.B. Ferrari, F.M. da Silva, L.A. Machado, C. Ramari, C.O. Brandão, L.M.B. dos Santos, L.L. dos Santos-Neto

**Affiliations:** 1 Departamento de Ciências Médicas, Universidade de Brasília, BrasíliaDF Brasil Departamento de Ciências Médicas, Universidade de Brasília, Brasília, DF, Brasil; 2 Instituto de Biologia, Universidade Estadual de Campinas, Departamento de Genética, Evolução, Microbiologia e Imunologia, Unidade de Neuroimunologia, CampinasSP Brasil Departamento de Genética, Evolução, Microbiologia e Imunologia, Unidade de Neuroimunologia, Instituto de Biologia, Universidade Estadual de Campinas, Campinas, SP, Brasil; 3 Hospital de Base de Brasília, Departamento de Psicologia, BrasíliaDF Brasil Departamento de Psicologia, Hospital de Base de Brasília, Brasília, DF, Brasil

**Keywords:** Neurofilaments, Depression, Anxiety, Multiple sclerosis

## Abstract

There is increasing evidence that neurofilament light chain (NF-L) can be considered as a biomarker for neuro-axonal damage. This polypeptide can be released into the cerebrospinal fluid (CSF) and the blood, where it can be quantified. The concentration of NF-L is elevated in patients with multiple sclerosis (MS) and psychiatric disorders. We aimed to investigate the NF-L levels in the CSF from treated MS patients and the relationship with depression or anxiety. The study involved three groups: control group (individuals without inflammation), the relapse-remitting multiple sclerosis (RRMS)-untreated group, and the RRMS-Fingo group (RRMS patients who were treated with fingolimod). MS disability was assessed by the Expanded Disability Status Scale, and depression and anxiety were evaluated by a neuropsychologist, using the Hospital Anxiety and Depression Scale, the Beck Depression Inventory-II, and the Beck Anxiety Inventory. Individual CSF samples were collected to measure NF-L levels. The results of the statistical analysis on levels of NF-L in the CSF of control subjects, RRMS-untreated patients, and RRMS-Fingo patients were significant. The relationship between depression and anxiety in RRMS-Fingo patients and NF-L levels was not statistically significant. In conclusion, MS events such as anxiety and depression appear to contribute to the onset of clinical relapses, subclinical cases, and neurodegeneration.

## Introduction

Multiple sclerosis (MS) is the most frequent disabling neurological disease in young adults, except for those of traumatic causes (1,2). The disease is a consequence of inflammation and neurodegeneration in the central nervous system (CNS). Relapse-remitting multiple sclerosis (RRMS) is the most frequent form of MS. Similar to other autoimmune diseases, the pathophysiology of MS involves genetic and environmental factors (3). However, events such as psychological stressors and states of anxiety and depression in RRMS also appear to contribute to the onset of clinical relapses and subclinical cases (4). New gadolinium-containing inflammatory lesions observed via magnetic resonance imaging (MRI) can be interpreted as biomarkers of unfavorable disease evolution, which includes motor, sensory, and cognitive impairments (5).

### MS and neurofilaments

The most promising biomarkers for neurodegeneration in MS and in other degenerative diseases are neurofilaments light chain (NF-L). Neurofilaments are important parts of the cyto-axonal cell structure as they constitute a major component of the axon cytoskeleton (5,6). There is increasing evidence that neurofilaments can be regarded as biomarkers for neuro-axonal damage, as axonal destruction results in disintegration of the axon membrane, neurofilament breakdown, and the subsequent release of neurofilaments into the cerebrospinal fluid (CSF), where they can be measured. Neurofilaments are subdivided into light, medium, and heavy chains (NF-H) according to their size (6). Elevated levels of these proteins have been interpreted as axonal damage and neuronal death in MS, Alzheimer's disease (AD), frontotemporal dementia (FTD), and motor neuron diseases (5). In MS, NF-L may be considered as an indicator in the CSF for disease activity. NF-L has been regarded as a possible prognostic marker for chronic disability, as measured by the Kurtzke Expanded Disability Status Scale (EDSS) (7). Both NF-H and NF-L seem to be associated with gadolinium-enhanced MRI lesion activity and recent findings indicate that an increase in serum NF-L may predict the appearance of such lesions (8). NF-L in the CSF might predict disease activity after the first demyelinating event suggestive of MS (9). However, the relationship between NF-L levels in the CSF and long-term disease progression has not yet been examined in detail (10).

Current studies correlate NF-L levels with the risk of conversion to MS after optic neuritis and treatment response to immunomodulatory drugs, such as fingolimod and natalizumab. These correlations indicate reduced axonal damage in patients switching from first-line disease-modifying drugs (DMDs) to fingolimod. NF-L levels in patients switching from natalizumab indicate similar effects on inflammatory and degenerative processes (11,12).

The therapeutic options for MS patients have been increasing in recent years, with a particular emphasis on oral drugs such as fingolimod. Mechanistically, fingolimod (Gilenya^®^, Novartis Pharma AG, Switzerland) binds to sphingosine 1-phosphate receptors on lymphocytes leading to retention of circulating lymphocytes in the lymph nodes. This reversible reduction in the number of peripheral blood lymphocytes is postulated to be mechanistically important in MS, decreasing the recirculation of autoreactive lymphocytes and preventing their infiltration into the CNS.

### Anxiety, depression, and neurofilaments

Recent studies also suggest that the mean concentration of NF-L is elevated in patients with bipolar disorders and other psychiatric disorders compared with healthy controls (13,14). Additionally, treatment resistance to major depression was correlated with increased plasma levels of NF-L, reflecting axonal damage (15,16).

As a marker of axonal damage, NF-L levels are elevated early in many neuropsychiatric disorders. This correlates with disease progression and brain atrophy in AD, MS, FTD, and amyotrophic lateral sclerosis, among others (15,17).

This study aimed to investigate the NF-L levels in the CSF of MS patients treated with fingolimod and the relationship with depression or anxiety.

## Material and Methods

The inclusion criteria were: 1) aged 18 years or older; 2) diagnosis of RRMS according to the McDonald criteria (18); 3) relapse-free over the past 30 days; and 4) mild MS disability as evidenced by a rating on the EDSS (7).

The exclusion criteria were: 1) inability to understand the motor test commands; 2) non-controlled chronic medical conditions, such as hypertension, diabetes, and cardiac conditions; and 3) other neurologic conditions in addition to MS.

To evaluate neurodegeneration, patients from the Brasília District Hospital and University of Campinas were divided into three groups. The control group contained individuals with tension-type headache, without inflammatory diseases. Because of ethical issues in collecting CSF of healthy individuals, we recruited volunteers with no symptoms of inflammation who were going to collect CSF for diagnostic purposes. After confirmation of tension-type headache, we utilized the CSF samples of these individuals as the control group. Patients not receiving any type of DMDs composed the RRMS-untreated group. The majority of these patients had a recent diagnosis (less than a year), mild symptoms, and were attending a follow-up medical appointment. A few of these patients with longer disease time and mild symptoms had refused to receive DMDs. The last group was formed by RRMS patients treated with fingolimod. These patients were recruited because they showed some degree of depression, probably due to the complex mechanisms involved in MS and not treatment-related.

### EDSS, Beck-II, BAI, and HADS scales

The attending physicians assessed the patient's level of disability using the EDSS and a neuropsychologist employed the following validated scales during the examination: the Hospital Anxiety and Depression Scale (HADS: anxiety subscale, HADS-A; depression subscale, HADS-D) (19,20), the Beck Depression Inventory-II (BDI-II) (21,22), and the Beck Anxiety Inventory (BAI) (23,24).

The degrees of depression and anxiety were based on the related scales' cutoff points as follows: no or minimal depression, BDI-II score of 0-11; mild depression, 12-19; moderate, 20-35; and severe, 36-63. The anxiety scale was similar. Absence of depression or a depression score of 0 and a BDI-II indicating depression or anxiety were scored as yes or no and the score was used to stratify the participants into two groups and simplify the statistical analysis. That is, patients who scored less than 18.5 points were considered as not having depression and patients with a score greater than or equal to 18.5 points were considered as having depression. In the HADS, the abnormal scores ranged from 11-21.

### Quantification of neurofilaments

Individual CSF samples were collected and centrifuged at 288 *g* at 4°C for 6 min. A 50-µL aliquot of the supernatant from each sample was used to measure NF-L levels by enzyme-linked immunosorbent assay (ELISA) following the manufacturer's instructions (Uman Diagnostics AB, Sweden).

### Ethical aspects

The study complied with the main national and international ethical regulations of research (REF Resolution CNS No. 466/2012 and Document of the Americas), registered in the National Commission of Ethics in Research (CONEP) through the Brazil platform and approved by the Ethics Committee of SES-DF (CAAE: 22477313.9.0000.5553/Opinion: 660.753). The consent process was obtained individually with all patients and controls having sufficient time to clarify their doubts and decide whether or not to participate in the study.

### Statistical analysis

Statistical analysis to compare NF-L levels was performed using GraphPad Prism 6 software (USA). Data are reported as median and range. Groups with P-values <0.05 were considered significantly different. The analysis of variance of the groups was performed by the Kruskal-Wallis test, followed by the Mann-Whitney test to compare the pair's medians.

## Results

**Figure 1:**
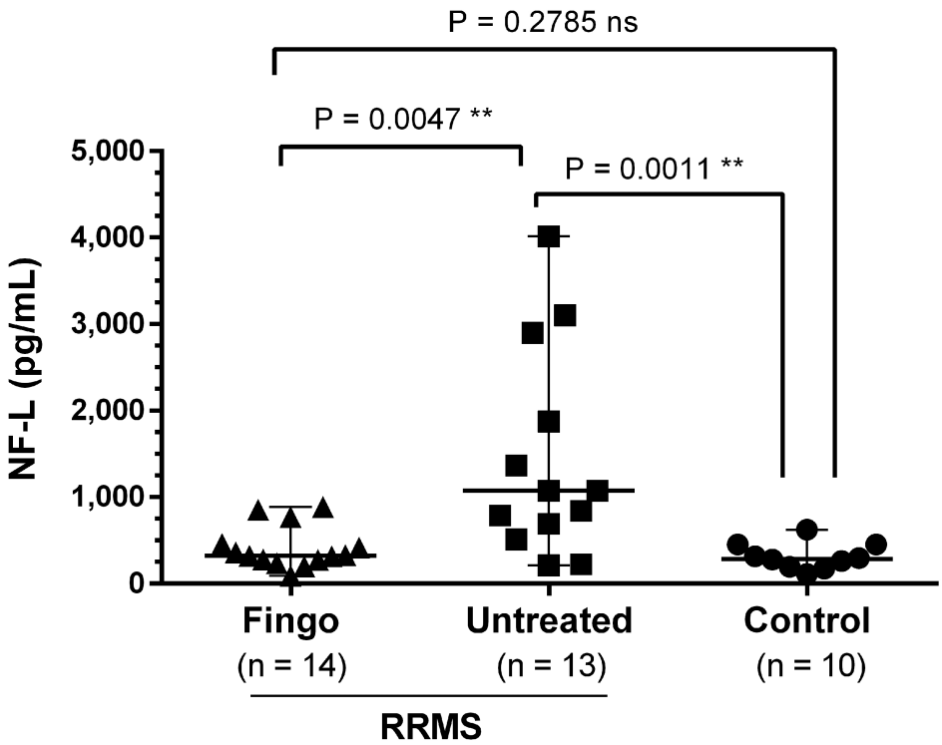
Level of neurofilaments light chain (NF-L) in the cerebrospinal fluid of control patients, untreated patients with relapse remitting multiple sclerosis (RRMS), and RRMS patients treated with fingolimod (Fingo) patients. Data are reported as median and range. Statistical analysis was carried out with the Kruskal-Wallis test, followed by the Mann-Whitney test. ns: not significant.

Patients’ demographic information is reported in Table 1. The analysis of NF-L levels is represented in Figure 1. RRMS patients treated with fingolimod showed concentrations of NF-L comparable to the control group, with the median values around 320 and 280 pg/mL, respectively. Whereas RRMS-untreated patients presented higher NF-L levels, ranging from 210 to 4015 pg/mL. Two of the RRMS untreated patients had been diagnosed less than three months before the sample collection. Outliers were excluded from these analysis.

**Table 1:** Demographics and clinical data of untreated patients with relapse remitting multiple sclerosis (RRMS), RRMS patients treated with fingolimod (Fingo), and controls.

	CSF		
	Control	RRMS-Untreated	RRMS-Fingo
Number of subjects	10	14	16
Gender (M:F)	1:2	5:9	3:13
Age in years (range)	38 (21-59)	33 (18-59)	42 (23-62)
Period under fingolimod medication, in years (range)	NA	NA	3 (0.6-5.5)
EDSS (range)	NA	2.2 (1-6.5)	2.4 (1.5-6.5)
Period after diagnosis, in years (range)	NA	3.8 (0.1-17)	7.9 (3.5-15)

EDSS: Expanded Disability Status Scale; NA: not applicable.

**Figure 2:**
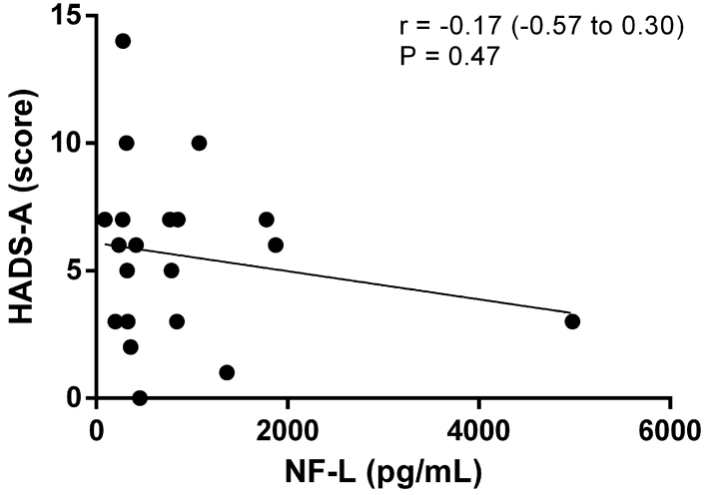
Correlation analysis between scores of the Hospital Anxiety and Depression Scale-anxiety (HADS-A) and neurofilaments light chain (NF-L) levels.

**Figure 3:**
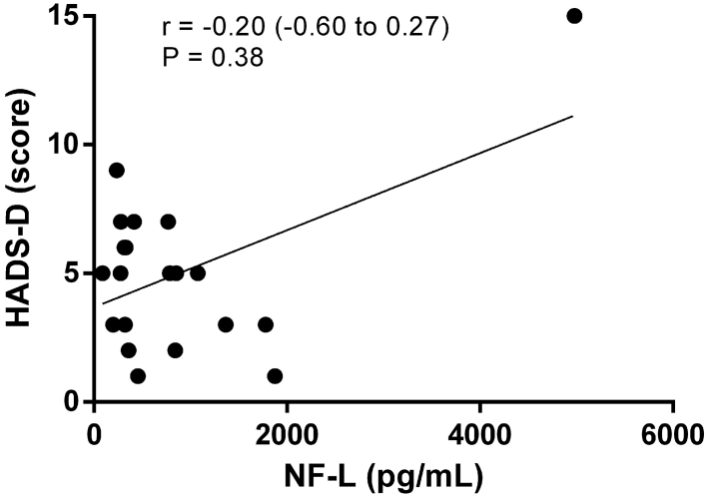
Correlation analysis between scores of the Hospital Anxiety and Depression Scale-depression (HADS-D) and neurofilaments light chain (NF-L) levels.

**Figure 4:**
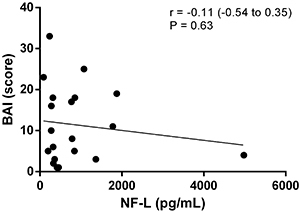
Correlation analysis between scores of the Beck Anxiety Inventory Scale (BAI) and neurofilaments light chain (NF-L) levels.

**Figure 5:**
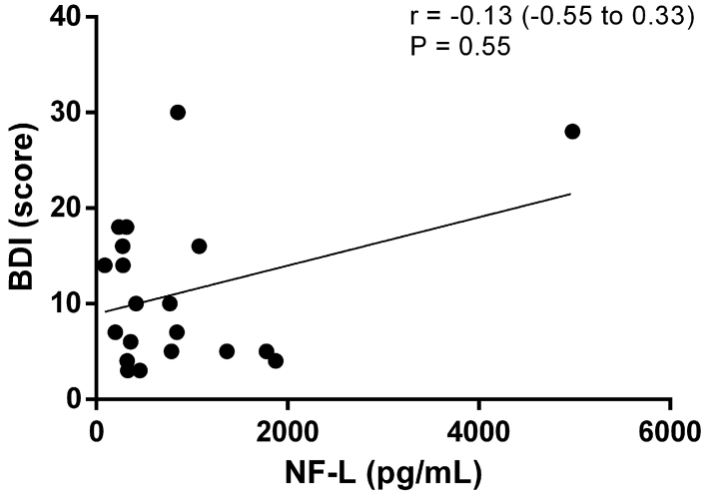
Correlation analysis between scores of the Beck Depression Inventory Scale (BDI) and neurofilaments light chain (NF-L) levels.

The relationship between depression and anxiety in MS patients using fingolimod and NF-L levels was not statistically significant, as demonstrated by the correlations between NF-L levels and the HADS and Beck scales. Outliers were not excluded (Figures 2, 3, 4, and 5).

## Discussion

We hypothesized that MS patients with anxiety and depression have a higher neurodegenerative activity and that this may increase the levels of NF-L in the CSF during the onset of the disease. Increased NF-L levels result in neurodegeneration caused by both MS injuries and mechanisms of depression. Furthermore, patients with both clinical conditions respond less to DMDs. Consequently, higher levels of NF-L in the CSF of these patients might be a predictive factor of therapeutic failure (25–28).

Recent studies suggested key aspects for the clinical follow-up of MS patients and that NF-L levels remain high in untreated patients even when they are not in relapse, which shows that the neurodegenerative processes continue (8–10). The other aspect is the reduction of NF-L levels in the CSF of patients receiving DMDs, which indicates the efficacy of medications against the neurodegenerative process (29). Real-world data show increasing evidence that NF-L levels are reduced after effective MS treatment (12,30). This has been demonstrated in patients receiving different types of treatment (10). Furthermore, a study conducted by Kuhle et al. (11) revealed similar results regarding NF-L levels although it was performed with patients who participated in fingolimod pivotal controlled clinical studies. The present study differs because it used real-world data from patients with and without MS treatment (31,32). The slightly higher levels of NF-L in the CSF of MS patients confirmed that axons probably continue to be damaged in these patients.

Multicenter studies are underway to consolidate neurofilaments as biomarkers that reflect brain tissue damage, enabling longitudinal monitoring of disease activity and drug effects in clinical trials of neurological diseases (11).

Factors associated with depression and anxiety disorders contribute to the worse evolution of neurodegeneration in MS (13,14,33). Jakobsson and collaborators assessed a large group of euthymic bipolar disorder patients and found elevated levels of NF-L in patients' CSF compared to healthy controls (13).

This paper aimed to study the relationship between NF-L levels and depression or anxiety in MS patients. As mentioned, analysis of NF-L levels might contribute to evaluate neurodegeneration during the course of MS (34–37). Our results are in accordance with the literature, because RRMS patients treated with fingolimod did show lower levels of NF-L than untreated patients. Therefore, we could assume that the duration of MS is longer in patients treated with fingolimod compared to untreated patients. However, at the moment, studies with neurofilaments have not shown significant statistical differences on this aspect.

One limitation of this study was the NF-L dosage in the CSF. Although De Flon et al. (38) showed that CSF NF-L level has a higher sensitivity, in this study specifically, blood NF-L concentration would probably be more appropriate. However, due to the lack of available technology in our country, this could not be performed. Another limitation of this study was the inclusion of only one form of MS, the RRMS, which is the most frequent in relation to other forms of MS. The reason for this was to provide a sample of convenience and the high cost of neurofilament technology for measurement in different groups.

The reason for the group using fingolimod as the only DMD was also for convenience. In addition, we had a particular interest in this group of patients since at the time in our outpatient cohort, we had MS patients using interferons, glatiramer acetate, natalizumab, and fingolimod. It turns out that fingolimod was the only DMD for oral use in contrast to the others that were injectable. The use of injectable medications can be interpreted as a bias due to the aspect of stress that can also influence the mood of patients. A final factor for the choice was the mechanism of action of fingolimod that causes the reduction of circulating lymphocytes, resulting in less pro-inflammatory activity of cytokines that could influence depression and an increase in other enzymes, such as idoleamine-2,3-dioxygenase, which reduce production of serotonin in the kynurenine pathway. Also, the action of other DMDs can also induce other mechanisms, such as the pro-inflammatory action of interferons or the blocking of the migration of lymphocytes through the blood-brain barrier to the central nervous system by natalizumab. However, the analysis of this stratification of patient groups by type of multiple sclerosis and disease-modifying therapy is of interest to us in many aspects and will be carried out in the next stages of this research.

The second purpose of the present study was to evaluate the relationship between depression and anxiety of MS patients treated with fingolimod and NF-L levels, which was not statistically significant. This may have been due to the small sample size in our study and the associated use of antidepressants. However, studies with larger and more homogeneous samples may provide evidence for a relationship between higher levels of NF-L in MS patients with depression and anxiety.

A final probable limitation of this study was the use of the HADS, BDI-II, and BAI scales to define the diagnosis of depression and anxiety. To minimize bias, we applied the scales and collected samples of CSF from patients at the same time. On the other hand, the diagnostic validation using structured scales was discussed in detail by Marrie et al. (39), concluding that these instruments have a reasonable performance and have statistical significance for reliable psychometric results in the definition of depression and anxiety among MS patients. A previous study by our group came to similar conclusions about the BDI-II scale (40).

The presented preliminary results did not support the hypothesis that NF-L levels in patients with depression or anxiety and MS are higher. Another conclusion from this study is that MS patients treated with fingolimod might have the neurodegenerative process controlled, since NF-L levels were comparable to the non-inflammatory control group.
